# VIEWDEX 3.0—RECENT DEVELOPMENT OF A SOFTWARE APPLICATION FACILITATING ASSESSMENT OF IMAGE QUALITY AND OBSERVER PERFORMANCE

**DOI:** 10.1093/rpd/ncab014

**Published:** 2021-03-04

**Authors:** Angelica Svalkvist, Sune Svensson, Tommy Hagberg, Magnus Båth

**Affiliations:** Department of Medical Physics and Biomedical Engineering, Sahlgrenska University Hospital, Gothenburg SE-413 45, Sweden; Department of Radiation Physics, Institute of Clinical Sciences, The Sahlgrenska Academy, University of Gothenburg Gothenburg SE-413 45, Sweden; Department of Medical Physics and Biomedical Engineering, Sahlgrenska University Hospital, Gothenburg SE-413 45, Sweden; Department of Medical Physics and Biomedical Engineering, Sahlgrenska University Hospital, Gothenburg SE-413 45, Sweden; Department of Medical Physics and Biomedical Engineering, Sahlgrenska University Hospital, Gothenburg SE-413 45, Sweden; Department of Radiation Physics, Institute of Clinical Sciences, The Sahlgrenska Academy, University of Gothenburg Gothenburg SE-413 45, Sweden

## Abstract

ViewDEX (Viewer for Digital Evaluation of X-ray Images) is an image viewer compatible with Digital Imaging and Communications in Medicine (DICOM) that has been especially designed to facilitate image perception and observer performance studies within medical imaging. The software was first released in 2004 and since then a continuous development has been ongoing. One of the major drawbacks of previous versions of ViewDEX has been that they have lacked functionality enabling the possibility to evaluate multiple images and/or image stacks simultaneously. This functionality is especially requested by researchers working with modalities, where an image acquisition can result in multiple image stacks (e.g. axial, coronal and sagittal reformations in computed tomography). In ViewDEX 3.0 this functionality has been added and it is now possible to perform image evaluations of multiple images and/or image stacks simultaneously, by using multiple monitors and/or multiple image canvases in monitors. Additionally, some of the previously available functionality has been updated and improved. This paper describes the recent developments of ViewDEX 3.0.

## INTRODUCTION

The introduction of digital radiography in the late 1900s resulted in new challenges regarding the optimization of image quality and radiation dose. Digital technology enables the possibility to change the appearance of a collected radiograph not only by changing the acquisition parameters, but also by adjusting the image processing. Hence, the task of optimising the examinations became more challenging as more variables were added to the equation. At many radiological departments one limiting factor in the optimization process is finding time for the radiologists to review and evaluate the radiological images. In order to facilitate the image evaluation process, the software ViewDEX (Viewer for Digital Evaluation of X-ray Images) was developed^(1)^. The software was first released in 2004 and the prerequisites for the development were that the software should be DICOM compatible, easy to use, freely available and be suitable for both imaging research and clinical optimisation. Since the first release of the software, continuous development has been ongoing and additional papers describing the developments have been published^(2–5)^. The ViewDEX software can be downloaded free of charge from www.vgregion.se/sas/viewdex, and in the end of October 2020 ViewDEX had been downloaded from over 40 countries and had received over 230 citations in scientific publications. ViewDEX has for example been used for studies in conventional projection radiography^(6)^, tomosynthesis^(7)^, computed tomography (CT)^(8)^, mammography^(9)^, magnetic resonance imaging (MRI)^(10)^, nuclear medicine imaging^(11)^, interventional imaging^(12)^ and ultrasound^(13)^.

The development of ViewDEX has always been focused on keeping up with the technological progress within the field of medical imaging. The goal of the development is to create a software that enables the observers to review study images in a surrounding that resembles the real clinical situation. The largest limitation with previous versions of ViewDEX is that they only support the possibility to include one single image or image stack in each case. Hence, the clinical benefits obtained by e.g. multi-planar reconstructions (MPRs) cannot be fully utilised during image review using ViewDEX, as the different reconstructions cannot be shown to the observer side-by-side. Additionally, performing alternative forced choice (AFC) studies^(14)^ is cumbersome, as each set of images has to be manually created before the study is configured in ViewDEX. Other limitations with ViewDEX 2.0 include that the log file may be difficult to work with and that data from physical measurements cannot automatically be saved in the log file.

In this paper, ViewDEX 3.0 will be presented. This new version of the software has been adapted to modern medical imaging, e.g. by the development of a new architecture that enables the possibility to simultaneously review multiple images/image stacks.

## GENERAL FUNCTIONALITY IN ViewDEX

In Svalkvist et al.^(5)^ the history of the development of ViewDEX since the first release in 2004 is presented, together with a detailed description of the available functionality of ViewDEX 2.0. To summarise, ViewDEX 2.0 can handle DICOM images from different modalities, for example conventional projection radiography, CT, single-photon emission CT, positron emission tomography, MRI and ultrasound. The cases included in a study can be displayed in a unique randomised order for each observer. During image review the observers are able to alter the image display properties e.g. by changing the window settings or zoom and pan the images. The observers can also perform physical measurements in the images, such as distance measurements, area measurements and measurements of mean pixel value in specific locations of an image. If a study is based on localisation of pathology the observers can make localization markings in the images and also answer questions connected to the markings made. It is also possible for the observer to write general notes regarding specific cases included in the study. The person responsible for study setup has full control over the conditions for image review and also has the possibility to log into a study and review (‘show mode’) or edit (‘edit mode’) the responses from each specific observer.

## ViewDEX 3.0

ViewDEX 3.0 is written in the Java programming language version 8 (but also works on later versions) and the code can run on any desktop system. The software is compatible with Windows 64, Linux 64 and MacOS 64. Compared to ViewDEX 2.0, version 3.0 is optimised with respect to performance, leading to for example shorter execution times.

### Multiple monitors and multiple canvases on each monitor

In the previous version of ViewDEX only one image/image stack at a time could be displayed to the observer. As a consequence, each case included in a study could only consist of one image/image stack (Figure ure1). Already in the past this limitation caused problems as many conventional X-ray examinations could include images from several different projection angles. Today more advanced imaging technologies result in examinations including several different image stacks, e.g. MPRs in CT and MRI. In the clinical situation, all the image/image stacks included in the examination contribute with information that is important for diagnosis. Hence, in order to resemble the clinical situation and take advantage of the increased amount of information obtained when multiple reconstructions, reformations and/or projections are available for diagnosis, it should be possible for each case included in a ViewDEX study to consist of more than one image or image stack, e.g. as illustrated in Figure 2.

**Figure 1:**
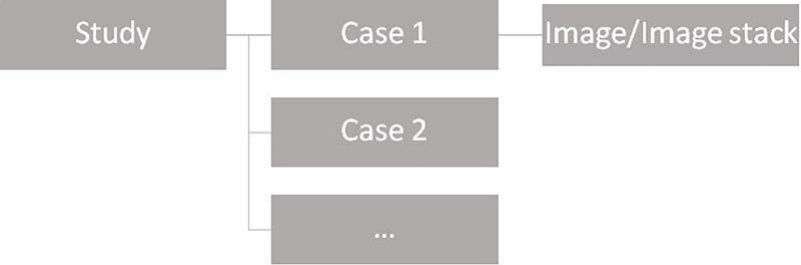
Schematic illustration of the configuration of the image database in ViewDEX 2.0. In ViewDEX 2.0 each case can consist of only one image or image stack.

**Figure 2:**
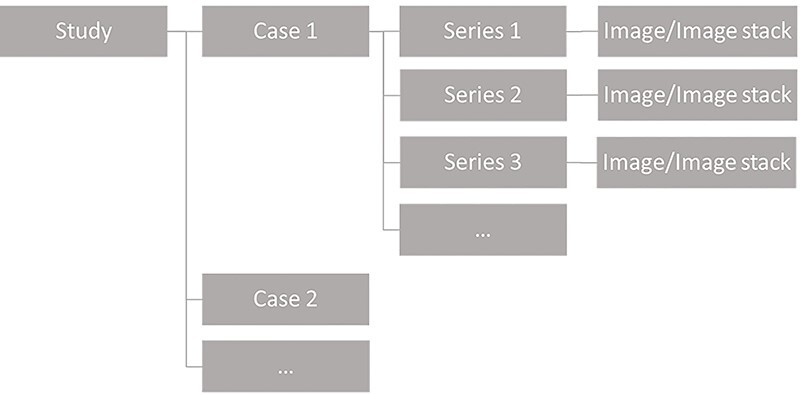
Schematic illustration of the configuration of the image database in ViewDEX 3.0. In ViewDEX 3.0 each case can consist of several different image series, each including either a single image or an image stack.

In order to facilitate the possibility to review multiple images simultaneously in ViewDEX, the architecture of the software has been changed. In ViewDEX 3.0, it is possible to review images using up to four different monitors. Additionally, each monitor can be divided into four separate image canvases, which means that a total of 16 different images/image stacks can be included in each case and reviewed simultaneously. Possible canvas setup on each monitor is 1 × 1 (full-screen), 1 × 2 or 2 × 1 (two canvases vertically or horizontally oriented) or 2 × 2 (four canvases). If three image/image stacks are included in each study and only one monitor is used, the canvas setup is 2 × 2 with one of the four canvases empty (black). Each image canvas will function as a separate display, which means that all functionality that was available in ViewDEX 2.0 is implemented in each canvas separately in ViewDEX 3.0. This means that the observer for example can change window settings and use zoom, pan and cine-loop individually in each image canvas. Additionally, the observers can mark two or more canvases, i.e. perform a multi-select of canvases. If more than one canvas is selected the scroll function (if image stacks are reviewed) and all changes of image properties (e.g. window settings, zoom and pan) are altered in the selected canvases simultaneously.

**Figure 3:**
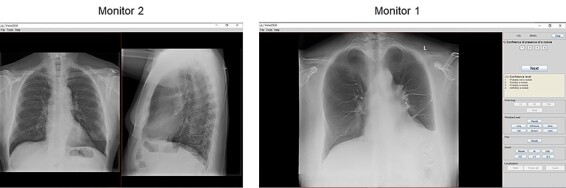
Screenshots of a study setup in ViewDEX 3.0. In this example, two monitors are used. The study consist of one chest tomosynthesis examination displayed on monitor 1 in full-screen (1 × 1) format (right) and two projections from a conventional chest examination (frontal + lateral) displayed side-by-side (1 × 2) on monitor 2 (left).

During the setup of a study the default configuration of number of monitors and number of canvases in each monitor is determined. During image review the observer has the possibility to temporarily change the image setup, e.g. to review one image/image stack in full-scale format on one of the monitors (1 × 1) or to review two out of four images on one monitor side-by-side (i.e. in canvas setup 1 × 2 or 2 × 1 instead of 2 × 2). In Figure 3, an example of a study setup using two monitors is presented. The case displayed consists of one chest tomosynthesis examination (image stack) and two projections from a conventional chest examination (frontal + lateral). The tomosynthesis examination is displayed in full-screen format (1 × 1) on Monitor 1, and the two projection images from the conventional chest examination are displayed side-by-side (1 × 2) on Monitor 2.

During study setup, the person responsible for study design specifies the location at which each image series should be displayed (monitor and canvas). However, it is also possible to choose that the location of each image series included in a case is randomised for each case and observer. This functionality enables the possibility to conduct AFC studies without any need to process the images in advance.

### Save results from physical measurements

Functionality enabling the possibility to perform physical measurements in images is a valuable tool in many image evaluation studies. Also in the clinic, measurements of different kinds are valuable in order to make a correct diagnosis. Already in ViewDEX 2.0 measurements such as distance, area and mean pixel values were enabled. This functionality has been used in many studies conducted using ViewDEX^(15–20)^. However, the fact that it is not possible to automatically store the results from physical measurements performed in ViewDEX 2.0 limits the usefulness of the functionality. Up until now, the only way to store results from physical measurements in ViewDEX has been to manually type the result in the notes panel. This solution comes with a number of disadvantages. For example, the risk for incorrect registration due to typing errors increases and if several different measurements are performed in each case, it may be difficult to distinguish the registered measurements from each other.

In ViewDEX 3.0, the observers can choose which measurements should be stored in the log file. A physical measurement is made by holding down specific buttons on the keyboard while clicking and dragging the mouse pointer over the image. By using keyboard short commando, the result from the measurement is stored and incorporated into the log file. For a distance measurement the distance will be stored in millimetres (mm). Additionally, the coordinates for the starting point and end point of the measured line will be given. For area measurements using a circular ROI or an ROI of variable shape, the measured area will be stored in cubic millimetres (mm^2^) and the mean pixel value will be given. The actual locations of the stored measurements can be reviewed in retrospect by logging in to the study in show mode.

### Restructured log file

The log file resulting from an image review study in ViewDEX is a text file including both information regarding the cases and the ratings of the observers for the tasks connected to the different cases included in the study. The information in the text file is separated using different delimiters. Unfortunately, many different delimiters are used in order to separate the data in the log file in ViewDEX 2.0. As a consequence, a lot of manual handling is necessary in order to for example extract the data to separate columns in e.g. Microsoft Excel. Due to both the new architecture in ViewDEX 3.0 and the possibility to store results from physical measurements, the log file needed a new structure. First, the amount of information stored in the log file is increased as each image/image stack included in one case may contain information stored during image review, e.g. localization markings or stored physical measurements. Therefore each case needs multiple lines in the log file in order to clearly display the resulting data. Second, the data included in the log file are now separated by only two types of delimiters, which makes it easier to separate the data into columns in e.g. Microsoft Excel.

## DISCUSSION

In order to thoroughly evaluate and/or optimise medical images, it is important to account for all aspects of an examination. One important part is to include all available information in the evaluation. If an examination results in more than one image/image stack all of these should be reviewed together. In previous versions of ViewDEX only one image/image stack at a time could be reviewed. In order to customise ViewDEX to modern medical imaging, the architecture of the software has been updated. In ViewDEX 3.0, it is possible to review more than one image/image stack at a time by using multiple monitors and/or multiple canvases on each monitor.

In ViewDEX, the person responsible for study setup has full control over the design of the study and can accommodate the viewing condition as appropriate. The study setup is customised by editing a study property file, which is a simple text file. During study setup, it can be determined which functionality the observers should be able to use during the review of the images. This can be of importance for example in situations, where the study design requires that the images are evaluated e.g. with a fixed window setting or a fixed zoom level. In these cases, the possibility for the observers to change window settings or zoom level can be prohibited in the property file. Other functionality that strengthens the outcome of a study is the fact that the cases included in a study can be presented in a unique randomised order for each observer and that the observers are unable to return to a previous case at a later time during image review. This prohibits the risk for biases due to e.g. changes in the observers’ thresholds or due to a reduction in diagnostic accuracy because of fatigue during image review^(21)^.

It is difficult to determine the reading times using ViewDEX as these are affected by e.g. type of study, number of images in each case (single images or image stacks) and number of tasks for the observer. In an estimation of reviewing times using ViewDEX, Håkansson et al.^(4)^ found that a receiver operating characteristics (ROC) study^(22)^ with single images is reviewed with a rate of ~150 cases/hour, whereas a free-response ROC study^(23)^ with image stacks of 60 images/case is reviewed with a rate of ~20 cases/hour. One reason for the relatively high image review rates is the fact that both image display and the registration of answers are made using the same software. The observer can concentrate solely on image evaluation, while the software automatically logs the answers connected to the current case. If separate systems would be used for image display and for recording answers, the observers themselves would need to keep track of the recorded answers in order to certify that the answers are registered for the correct case.

Even though the development of ViewDEX 3.0 has led to a more modern and general software, there is still room for further improvements. The development of ViewDEX has been focused on reducing the effort needed by the observers reviewing a study, because one of the most common limiting factors for conducting image review studies is finding time for the observers to review the images. However, study setup in ViewDEX is still quite cumbersome. Technically, study setup is easy, as it only requires editing of the property files that are simple text files. Nevertheless, as more and more functionality is added to the software the property files become longer and study setup more time consuming. Also, small errors in e.g. spelling or data that accidentally was left out in the editing of the property files might lead to the fact that the study cannot be run in ViewDEX. Finding the error in order to correct the input in the property file is time consuming and often leads to frustration. Future releases of ViewDEX 3.0 will include the development of a semi-automatic configuration menu in which the details concerning the study setup can be customised. The configuration menu will require less manual texting in order to edit the property file. Instead, the menu will consist of different headings under which available editing alternatives are presented, e.g. as check boxes. Another future development may include making the software even more general regarding the configuration of monitors and canvases in each monitor. In the current release of ViewDEX 3.0, there is a limitation regarding the maximum number of simultaneous monitors and canvases/monitor (maximum four monitors and four canvases/monitor), leading to a maximum of 16 images/image stacks that can be reviewed simultaneously.

The fact that ViewDEX is not server based leads to limitations regarding the possibilities to easily conduct a ViewDEX study with observers from different sites. Today, the only solution in this situation is to distribute one copy of the study to each site. Another limitation is that ViewDEX cannot communicate with the Picture archiving and communication system (PACS), which means that the person designing the study needs to copy the images that should be included in the study from the PACS system and insert them into a separate folder, which will be working as the image database for the study. This work can be quite cumbersome and time consuming. There are, however, several reasons why the development of ViewDEX has not been focused on creating a server-based software. For example, such a solution might require a higher level of data security in order to follow the recommendations of the General Data Protection Regulation. Additionally, if a study can be reviewed online, the observers have full access to the study from an arbitrary number of locations. This might limit the possibilities for the person responsible for the study to control the image reading conditions, e.g. type of monitor, monitor calibration and surrounding light environment, which also might lead to biases in the results from a study.

## CONCLUSION

ViewDEX 3.0 is a more modern version of the well-established and frequently used image evaluation software ViewDEX. The software has now been configured to facilitate review of multiple images/image stacks simultaneously, enabling the possibility to use ViewDEX for evaluations of more complex imaging modalities.

## FUNDING

This work was supported by grants from the Swedish state under the agreement between the Swedish government and the county councils, the ALF-agreement [ALFGBG-718111]; The Healthcare Committee, Region Västra Götaland (Hälso- och sjukvårdsstyrelsen) [VGFOUREG-932018]; and the Euratom research and training programme 2014–18 under grant agreement No. 755523 (MEDIRAD).
